# Actigraphy-Derived Sleep Is Associated with Eating Behavior Characteristics

**DOI:** 10.3390/nu13030852

**Published:** 2021-03-05

**Authors:** Rocío Barragán, Faris M. Zuraikat, Victoria Tam, Samantha Scaccia, Justin Cochran, Si Li, Bin Cheng, Marie-Pierre St-Onge

**Affiliations:** 1 Sleep Center of Excellence, Department of Medicine, Columbia University Irving Medical Center, New York, NY 10032, USA; rocio.barragan@uv.es (R.B.); fmz2105@cumc.columbia.edu (F.M.Z.); ses2272@cumc.columbia.edu (S.S.); jc5392@cumc.columbia.edu (J.C.); 2 Division of General Medicine, Department of Medicine, Columbia University Irving Medical Center, New York, NY 10032, USA; 3 Department of Preventive Medicine and Public Health, School of Medicine, University of Valencia, 46010 Valencia, Spain; 4 CIBER Fisiopatología de la Obesidad y Nutrición, Instituto de Salud Carlos III, 28029 Madrid, Spain; 5 Division of Cardiology, Department of Medicine, Columbia University Irving Medical Center, New York, NY 10032, USA; 6 Institute of Human Nutrition, College of Physicians and Surgeons, Columbia University Irving Medical Center, New York, NY 10032, USA; bvt2104@cumc.columbia.edu; 7 Mailman School of Public Health, Columbia University Irving Medical Center, New York, NY 10032, USA; sl4657@cumc.columbia.edu (S.L.); bc2159@cumc.columbia.edu (B.C.)

**Keywords:** BMI, eating behavior, restraint, disinhibition, hunger, sex, sleep

## Abstract

Poor sleep is a determinant of obesity, with overconsumption of energy contributing to this relationship. Eating behavior characteristics are predictive of energy intake and weight change and may underlie observed associations of sleep with weight status and obesity risk factors. However, relationships between sleep and dimensions of eating behavior, as well as possible individual differences in these relations, are not well characterized. Therefore, the aim of this study was to evaluate whether sleep behaviors, including duration, timing, quality, and regularity relate to dietary restraint, disinhibition, and tendency towards hunger and to explore whether these associations differ by sex. This cross-sectional study included 179 adults aged 20–73 years (68.7% women, 64.8% with BMI ≥ 25 kg/m^2^). Sleep was evaluated by accelerometry over 2 weeks. Eating behavior dimensions were measured with the Three-Factor Eating Questionnaire. Prolonged wake after sleep onset (WASO) (0.029 ± 0.011, *p* = 0.007), greater sleep fragmentation index (0.074 ± 0.036, *p* = 0.041), and lower sleep efficiency (−0.133 ± 0.051, *p* = 0.010) were associated with higher dietary restraint. However, higher restraint attenuated associations of higher WASO and sleep fragmentation with higher BMI (*p*-interactions < 0.10). In terms of individual differences, sex influenced associations of sleep quality measures with tendency towards hunger (*p*-interactions < 0.10). Stratified analyses showed that, in men only, higher sleep fragmentation index, longer sleep onset latency, and lower sleep efficiency were associated with greater tendency towards hunger (β = 0.115 ± 0.037, *p* = 0.003, β = 0.169 ± 0.072, *p* = 0.023, β = −0.150 ± 0.055, *p* = 0.009, respectively). Results of this analysis suggest that the association of poor sleep on food intake could be exacerbated in those with eating behavior traits that predispose to overeating, and this sleep-eating behavior relation may be sex-dependent. Strategies to counter overconsumption in the context of poor quality sleep should be evaluated in light of eating behavior traits.

## 1. Introduction

There is increasing evidence that sleep influences cardiometabolic health, including obesity risk. Associations of short, poor, and variable sleep with obesity and its risk factors have been reported [1,2,3,4,5,6,7]. Overconsumption of energy is likely the primary behavioral driver of this sleep-obesity relation, as research findings consistently report increases in energy intake in response to reduced sleep [8,9,10].

A number of mechanisms have been proposed to explain the sleep-energy intake relation, including changes in appetite-regulating hormones and increases in hedonic responses to food stimuli [5]. However, increased reward valuation of food has been brought forth as the most likely driver of the rise in energy intakes following short sleep [11]. The association between the hedonic system and short sleep duration has been supported by neuroimaging studies showing an increase in the neuronal responses to food stimuli in regions of the brain related to the reward system and involved in hedonic feeding following sleep deprivation [12,13,14,15,16]. These findings indicate that underpinnings of the sleep-obesity relation are biopsychosocial.

Indeed, the regulation of food intake is complex, as it is influenced not just by physiologic factors, but also by cognitive, emotional, and behavioral factors [17,18]. Three key dimensions of human eating behavior are dietary restraint, disinhibition, and tendency towards hunger [19,20], hereafter referred to as hunger. The current literature reports associations between these components of eating behavior and obesity in adults [21,22,23]. Higher disinhibition is consistently related to higher body mass index (BMI) and has been shown to relate to higher energy intake as well [21,22,24,25,26,27,28,29,30]. In contrast, the relation between dietary restraint and weight-related outcomes is more complex. While increases in dietary restraint can improve diet quality [22,24,29,31,32] and lead to favorable weight loss outcomes [24,33], high dietary restraint has also been shown to have counter-regulatory effects on intake [34] and increases in restraint could be a result of weight gain or obesity. Finally, higher hunger may predispose individuals to weight gain and predicts poorer weight loss [35], as this characteristic is often associated with higher energy intake [24,36,37,38]. Evaluating the relations between sleep and dietary restraint, disinhibition, and hunger may help to better understand why individuals overconsume in response to short sleep.

To date, few studies have explored the possible relationship between sleep patterns and eating behavior characteristics with equivocal results. Poor sleep quality, measured using the Pittsburgh Sleep Quality Index (PSQI), has been associated with greater hunger, disinhibition and restraint in healthy adults with a history of diabetes [39]. Recent studies, however, failed to observe an association of self-reported sleep quality with restraint but rather observed associations with hunger and disinhibition [19,40]. These discrepancies may be due to participant characteristics, such as sex [19,28,38,41,42,43]. Indeed, we have shown that males and females have different physiological responses in appetite-regulating hormones in response to sleep restriction, whereby men display increased levels of ghrelin and women have reduced levels of glucagon-like peptide 1 following sleep restriction relative to adequate sleep [44]. Inconsistent findings across studies may also reflect limitations of subjective measures of sleep quality, highlighting the need for objective determinations of sleep patterns. The current study aims to comprehensively evaluate associations of actigraphy-derived sleep with eating behavior characteristics and explore whether sex influences these associations. We hypothesize that undesirable sleep patterns will be associated with lower dietary restraint, higher disinhibition, and greater hunger and that these patterns will be enhanced in men relative to women, given evidence that men overconsume to a greater extent in response to unhealthy sleep [45].

## 2. Materials and Methods

### 2.1. Study Population

**Figure 1:**
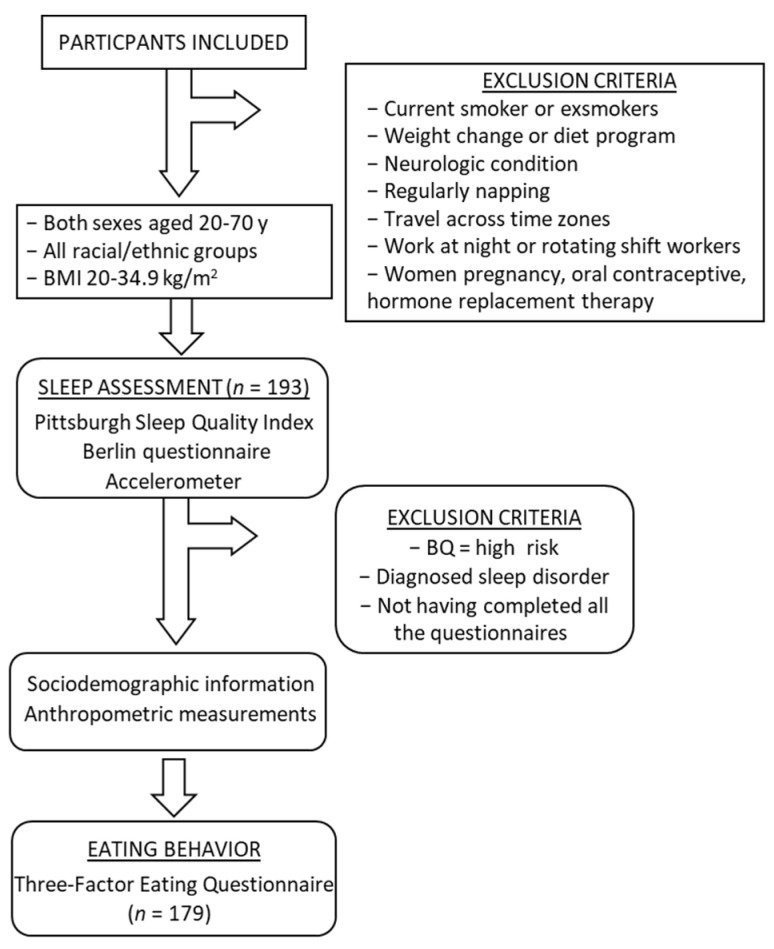
Flow chart of participant inclusion and exclusion criteria for the different measures included in the study. BMI: body mass index; PSQI: Pittsburgh Sleep Quality Index; BQ: Berlin Questionnaire.

This study was performed in 179 racially/ethnically diverse adults aged ≥20 year and with a BMI of 20–34.9 kg/m^2^ that met initial eligibility criteria to participate in studies related to sleep and circadian alignment at Columbia University Irving Medical Center between 2016 and 2020 (NCT02960776, NCT02835261, NCT03663530). Individuals interested in participating in these trials contacted the laboratory and completed a preliminary phone screening. Exclusion criteria evaluated at this initial screening included current smoking or ex-smokers <3 year, and those with recent weight change or who actively participated in a diet or weight loss program in the previous 3 mo (Figure 1). Those with a neurologic condition deemed to potentially disrupt or interfere with the procedures were excluded as were individuals who regularly napped, traveled across time zones, or worked non-traditional hours (i.e., night or rotating shift work). Women who were pregnant, within 1 y post-partum, or on oral contraceptive or hormone replacement therapy were also excluded. Following the phone screening, potentially eligible individuals were invited to the laboratory for an in-person screening visit. Informed consent was obtained at the beginning of the in-person screening, prior to collecting any measures. Anthropometric measurements were then obtained to determine whether individuals met inclusion criteria for BMI. Participants also provided sociodemographic information and completed a variety of questionnaires to evaluate eligibility for the clinical trials. These questionnaires included a health history questionnaire as well as the Berlin Sleep Apnea scale [46], the Pittsburgh Sleep Quality Index [47], the Morningness-Eveningness Questionnaire [48], and the Three Factor Eating Questionnaire (TFEQ) [20].

### 2.2. Sleep Assessment

Participants who successfully cleared all of the inclusion and exclusion criteria described above (*n* = 193) were provided an accelerometer (GT3X+, Actigraph Corp, Pensacola, FL, USA) to assess their sleep over a 2-week period in order to assess sleep-related eligibility status for the study for which they screened (NCT02960776, NCT02835261, NCT03663530). The data obtained from the sleep measurement included total sleep duration (min), bedtime (time), waketime (time), midpoint of sleep (time), sleep efficiency (%), wake after sleep onset (WASO, min) and sleep fragmentation index. In addition, variability in bedtime and midpoint of sleep (sleep timing) were quantified for each participant by calculating the standard deviation (SD) of those measures across nights. Actigraphy data were scored in one-minute epochs and bedtime and waketime information was supplemented from sleep diaries.

### 2.3. Three-Factor Eating Questionnaire

Eating behavior characteristics were assessed using the TFEQ [20]. The questionnaire consists of 51 items and measures three dimensions of human eating behavior: (1) restraint, or cognitive control of food intake (21 items); (2) disinhibition, or tendency to overeat due to a loss of control over food intake (16 items); (3) hunger, or susceptibility to perceptions or feelings of hunger (14 items). Each item scores 0 or 1, for a maximum score of 21 for restraint, 16 for disinhibition, and 14 for hunger. Higher scores indicate higher levels of dietary restraint, more disinhibited eating, and greater predisposition towards hunger. All participants completed the TFEQ on the day of or 1–2 days prior to the sleep assessment period.

### 2.4. Statistical Analyses

Of the 193 participants that took part in the sleep screening, 179 were included in the current analyses. Those excluded from the analyses had missing values (8 for race/ethnicity or education; 2 for TFEQ outcomes; 1 for several covariates) or outlier values (3 for standard deviation of bedtime and/or duration ≥240 min). Baseline characteristics of the analytic sample are summarized as Mean ± Standard Deviation (SD) for continuous variables or as count (percent) for categorical variables. Independent sample T-test and chi-squared test were used to evaluate mean differences in descriptive characteristics between men and women for continuous and categorical variables, respectively. Sleep characteristics, derived from actigraphy, were the exposure variables of interest: sleep duration, efficiency, and latency, WASO, sleep fragmentation index, bedtime and bedtime variability (defined as standard deviation (SD) of bedtime), and sleep timing (defined as midpoint of sleep episode). Outcomes of interest were scores for the TFEQ subscales: dietary restraint, disinhibition, and hunger. Linear regression models were used to evaluate associations of sleep variables with TFEQ subscales, both on the continuous scale. Each sleep exposure was evaluated individually with each TFEQ outcome variable. The first models (model 1) evaluated univariate associations between exposure and outcome variables. Models were then adjusted for potential confounders: age, sex, race/ethnicity and education (model 2). We then evaluated whether sex influenced relations of sleep with TFEQ subscales by assessing the interaction between sleep and sex. In separate analyses, we evaluated the interaction of sleep with eating behavior characteristics on the outcome of BMI to explore whether established associations of sleep with BMI [6,49,50] were influenced by eating behavior characteristics. Due to limited power, this was only assessed in the full sample and not by sex. The R software version 3.6.1 was used for statistical analyses. Results of regression models are presented as β ± SE and were considered significant at *p* < 0.05 for main effects and <0.10 for interaction effects given the exploratory nature of these analyses and limited sample size.

## 3. Results

### 3.1. Participants Characteristics

The demographic, health, and sleep and eating behavior characteristics of the sample are shown in Table 1. Of the 179 participants included in analyses, 68.7% were women and 64.8% had body mass index (BMI) ≥ 25 kg/m^2^. Average age of the sample was 36.0 ± 13.1 year. Women were older, had higher educational attainment, and had earlier wake time and midpoint of sleep than men. No differences were found in eating behavior characteristics between men and women. We also analyzed the baseline descriptive characteristics between participants with and without overweight/obesity (BMI ≥ 25 kg/m^2^). Participants with overweight were older, had later bedtime and WASO, and had a higher restraint and disinhibition scores on the TFEQ (Table S1).

### 3.2. Associations of Sleep Parameters with TFEQ Items

In the full sample, actigraphy-derived continuous measures of WASO and sleep fragmentation index were positively associated with dietary restraint (*p* = 0.007 and 0.041, respectively; Table 2). In addition, poorer sleep efficiency was associated with higher dietary restraint (*p* = 0.010). Although we did not find statistically significant associations between sleep and disinhibition or hunger traits, we observed trends for associations between lower sleep efficiency (*p* = 0.076) and higher WASO (*p* = 0.085) and greater tendency towards hunger. Sleep duration and sleep timing-related variables were not associated with eating behavior characteristics in this sample.

### 3.3. Evaluation of Inter-Individual Differences in Relations of Sleep with TFEQ Constructs

Assessment of individual differences revealed sex specific associations of sleep parameters with eating behavior characteristics. Participant sex significantly influenced associations of sleep onset latency and sleep fragmentation index with hunger, whereby associations were observed in men (both *p* < 0.05) but not in women (Table 3). We also noted a trend towards sex-specific associations of sleep efficiency with hunger (*p*-interaction = 0.098). Results of stratified analysis showed that higher sleep efficiency related to lower tendency towards hunger in men, with no association between these factors in women.

### 3.4. Evaluation of the Influence of Eating Behaviors on Associations between Sleep and BMI

Finally, we assessed whether associations between sleep characteristics and BMI were moderated by eating behavior characteristics. Results showed that higher dietary restraint attenuated the positive relationships between sleep fragmentation index and WASO with BMI (WASO: −0.003 ± 0.002; *p* = 0.071; sleep fragmentation index: −0.013 ± 0.006: *p* = 0.034) in models adjusted for confounding variables. In addition, higher dietary restraint made the slope of the association between sleep efficiency and BMI less negative (0.015 ± 0.0084, *p* = 0.076).

## 4. Discussion

This study provides novel results relating actigraphy-derived sleep behaviors to dimensions of eating behavior. We demonstrate sex-specific associations between multiple parameters of sleep quality with greater hunger, a characteristic previously been shown to predict blunted weight loss [35], with associations only observed in men. Notably, this result aligns with prior findings that ghrelin, a hunger-promoting hormone increases in men with insufficient sleep [44]. Moreover, we showed that dietary restraint moderated associations between sleep fragmentation and WASO and higher BMI, which could imply that individuals with high dietary restraint may be at lower risk for weight gain in response to poor sleep.

We identified consistent associations between components of sleep quality and hunger in men, indicating differences in the predisposition to overconsumption in response to poor sleep between men and women. These results further support prior findings [19,39,51,52]. Susceptibility to hunger has been directly associated with energy intake [28,38,53] and body weight [24,36,37,38], and the underlying mechanism may be due to an increased activity in areas related to food reward [22,54,55,56] as well as hormonal regulation of appetite. This association between sleep quality and hunger trait supports observations that sleep restriction promotes activation of brain neuronal networks involved in interoception [16], and increases ghrelin levels in men but not in women [44]. Taken together, these findings further explain higher energy intake [45] and lower subjective ratings of fullness [10] detected among men in sleep restriction conditions.

In our cohort, no association was detected between sleep measures and disinhibition, as other studies have noted [57,58]. In contrast, other authors related associations between poorer self-reported sleep quality and higher disinhibition [19,39,51,52]. Blumfield et al. showed that disinhibition trait mediated the relationship between sleep quality and weight status [19]. Conversely, a similar analysis by Chaput et al. failed to show this association between sleep and this eating behavior [57], in agreement with the findings of our study. However, in longitudinal analyses, individuals with short sleep duration gained weight over a 6-year follow-up period only if they had high disinhibition [57]. These findings highlight limitations of cross-sectional observations and demonstrate the need for further longitudinal and clinical investigations into potential influence of eating behaviors in the role of sleep on energy balance.

Finally, we found that poor sleep outcomes were related to greater dietary restraint. To date, few studies have evaluated relations of sleep with dietary restraint, and as mentioned above, the results have been diverse [19,39,51]. Higher levels of cognitive restraint have been associated with consumption of a higher quality diet and lower energy intake [28,31,32,38,53,59,60,61], but have also been related with higher energy intake [62,63], lower intake of organic food [64], or not associated with dietary intake [29,65]. Neuroimaging studies have shown that those with high restraint scores show hyper-response in brain areas related to food reward when presented with food images or in the presence of food [66,67]. Our observations showing that poor sleep outcomes are related to greater restraint may reflect this propensity towards hyper-responsivity to food in the context of poor sleep, which may also increase reward salience of foods. Unfortunately, prior studies have not reported on eating behavior traits of participants and this hypothesis remains untested. However, we could also speculate that dietary restraint may be practiced to prevent overeating in response to hypersensitivity to food reward following poor sleep, thereby preventing weight gain in these individuals. This speculation is supported by our models suggesting that having greater restraint dampens the association between poor sleep and high BMI. It could also help to explain the large inter-individual variability in overeating in response to sleep restriction [68].

Our study has some notable strengths and limitations. The study design includes valid and reliable measures of sleep, using 2-week wrist actigraphy data, and eating behavior traits obtained through a validated questionnaire (TFEQ). In addition, moderation analyses provided an integral model that includes the three variables studied (eating behavior, sleep and BMI) to examine behavioral mechanisms linking sleep with obesity risk. On the other hand, our sample size is relatively small and participants were not randomly selected. All participants responded to advertisements for research studies that sought individuals with adequate sleep. As a result, although some who screened failed screening due to poor sleep duration were included in the present analyses, those with overt sleep disorders were not included. However, despite this selection requirement, our sample still provided a wide range of sleep durations and quality. Moreover, our study includes racially diverse participants of both sexes, with wide age and BMI ranges. Finally, being a cross-sectional study, we cannot infer causal effects of sleep and eating behaviors on BMI. For this reason, it is necessary to carry out longitudinal and experimental studies that replicate the results obtained in our population.

## 5. Conclusions

In conclusion, this study provides evidence that poor sleep patterns and eating behavior traits are correlated. For the first time, we provide evidence of sex-specific associations between poor sleep and tendency towards hunger. Particularly in men, differences in eating behavior traits may underlie susceptibility to overeating in response to poor sleep. These data may explain inter-individual variability in food intake in response to poor sleep and may inform strategies to target weight management efforts in those with poor sleep and obesity. For example, strategies to curb susceptibility to hunger in sleep apnea, a condition where sleep fragmentation is a hallmark feature, or insomnia, marked by difficulty initiating sleep, may be particularly efficacious for weight management in men. Capitalizing on strengthening cognitive control of food intake may also be of importance for prevention of weight gain in fragmented sleep conditions.

## Figures and Tables

**Table 1:** Baseline descriptive characteristics of the overall analytic sample and by sex.

	Female (*N* = 123)	Male (*N* = 56)	Total (*N* = 179)	*p*-Value
**Demographic**				
**Age (years)**				
**Mean ± SD**	37.6 ± 14.2	31.5 ± 9.0	36.0 ± 13.1	0.004
**Range**	20.0–70.0	19.0–73.0	19.0–73.0	
**Race/Ethnicity**				
**White/Non-Hispanic**	93 (75.6%)	37 (66.1%)	130 (72.6%)	0.185
**Non-white/Hispanic**	30 (24.4%)	19 (33.9%)	49 (27.4%)	
**Education**				
**<College degree**	33 (26.8%)	25 (44.6%)	480 (32.4%)	0.018
**≥College degree**	90 (73.2%)	31 (55.4%)	121 (67.6%)	
**Health and Sleep Behaviors**				
**Body mass index (BMI) (kg/m^2^)**				
**Mean ± SD**	26.6 ± 3.6	26.4 ± 3.0	26.6 ± 3.4	0.737
**Range**	20.4 ± 34.9	17.4 ± 33.2	17.4 ± 34.9	
**Sleep duration**				
**Mean ± SD**	428.7 ± 46.1	423.6 ± 50.4	426.1 ± 47.4	0.512
**Range**	204.9–544.9	312.9–553.1	204.9–553.1	
**Bedtime**				
**Mean ± SD**	0:44 ± 5:06	1:41 ± 5:37	1:09 ± 5:24	0.154
**Range**	20:11–2:38	22:13–2:49	20:10–2:49	
**Waketime**				
**Mean ± SD**	7:42 ± 1:10	8:24 ± 1:13	7:55 ± 1:13	<0.001
**Range**	3:57–10:40	6:21–11:03	3:57–11:03	
**Midpoint of sleep**				
**Mean ± SD**	3:50 ± 0:56	4:26 ± 1:01	4:01 ± 0:59	<0.001
**Range**	1:23–6:40	2:32–6:59	1:23–6:59	
**Sleep efficiency (%)**				
**Mean ± SD**	88.4 ± 5.8	86.9 ± 6.4	87.9 ± 6.0	0.132
**Range**	67.0–97.1	68.7–96.4	67.0–97.1	
**Wake after sleep onset (min)**				
**Mean ± SD**	51.0 ± 28.1	57.0 ± 28.7	52.9 ± 28.3	0. 832
**Range**	10.3–175.8	13.9–141.4	10.3–175.8	
**Sleep fragmentation index**				
**Mean ± SD**	24.4 ± 7.9	28.4 ± 9.4	27.0 ± 8.4	0.132
**Range**	8.1–49.4	10.8–54.7	8.1–54.7	
**Midpoint SD (min)**				
**Mean ± SD**	52.1 ± 30.2	51.5 ± 29.5	51.9 ± 29.9	0.891
**Range**	4.0–201.2	11.6–140.6	4.0–201.2.7	
**Bedtime SD (min)**				
**Mean ± SD**	60.0 ± 33.7	62.4 ± 38.5	60.8 ± 35.2	0.669
**Range**	0.0–237.4	8.4–240.0	0.0–240.0	
**Eating Behavior Characteristics**				
**Dietary restraint**				
**Mean ± SD**	7.4 ± 4.1	7.7 ± 4.0	7.5 ± 4.0	0.684
**Range**	0.0–19.0	0.0–17.0	0.0–19.0	
**Disinhibition**				
**Mean ± SD**	3.9 ± 2.9	3.2 ± 2.1	3.7 ± 2.7	0.083
**Range**	0.0–13.0	0.0–8.0	0.0–13.0	
**Tendency towards hunger**				
**Mean ± SD**	2.9 ± 2.2	3.3 ± 2.6	3.0 ± 2.4	0.274
**Range**	0.0–11.0	0.0–13.0	0.0–13.0	

Values are mean ± SD for continuous variables and count (%) for categorical variables. BMI: body mass index; *p*-value for the comparisons (means or %) between men and women. Student’s t test was used to compare continuous variables and Chi squared tests were used to compare categorical variables.

**Table 2:** Cross-sectional analysis of measures of day-to-day sleep variability with eating behavior traits.

Predictor	Outcome	β ± SE ^a^ (Model 1)	*p*-Value (Model 1)	β ± SE ^b^ (Model 2)	*p*-Value (Model 2)
**Sleep duration**	Dietary restraint	−0.009 ± 0.006	0.134	−0.007 ± 0.007	0.2797
	Disinhibition	−0.002 ± 0.004	0.657	−0.000 ± 0.004	0.9308
	Tendency towards hunger	−0.004 ± 0.004	0.297	−0.005 ± 0.004	0.2000
**Bedtime**	Dietary restraint	0.420 ± 1.346	0.756	0.258 ± 1.331	0.847
	Disinhibition	−0.288 ± 0.892	0.747	−0.188 ± 0.876	0.830
	Tendency towards hunger	−1.074 ± 0.781	0.171	−1.187 ± 0.787	0.133
**Wake time**	Dietary restraint	5.316 ± 5.877	0.367	9.379 ± 6.113	0.127
	Disinhibition	−2.589 ± 3.899	0.507	1.751 ± 4.005	0.558
	Tendency towards hunger	2.811 ± 3.428	0.413	2.149 ± 3.661	0.558
**Midpoint**	Dietary restraint	3.838 ± 7.312	0.600	7.732 ± 7.567	0.308
	Disinhibition	−2.178 ± 4.847	0.654	2.909 ± 4.990	0.561
	Tendency towards hunger	5.883 ± 4.244	0.167	5.766± 4.50	0.202
**WASO**	Dietary restraint	0.030 ± 0.010	0.005	0.029 ± 0.011	0.007
	Disinhibition	0.000 ± 0.007	0.955	5.922 × 10^−5^ ± 7.191 × 10^−3^	0.993
	Tendency towards hunger	0.001 ± 0.007	0.122	0.011 ± 0.006	0.085
**Sleep efficiency**	Dietary restraint	−0.139 ± 0.051	0.006	−0.133 ± 0.051	0.010
	Disinhibition	0.015 ± 0.034	0.662	0.020 ± 0.034	0.5624
	Tendency towards hunger	−0.047 ± 0.029	0.113	−0.055 ± 0.031	0.076
**Sleep onset latency**	Dietary restraint	0.087 ± 0.052	0.097	0.067 ± 0.051	0.198
	Disinhibition	−0.019 ± 0.035	0.593	−0.030 ± 0.034	0.381
	Tendency towards hunger	0.032 ± 0.031	0.291	0.031 ± 0.031	0.319
**Sleep fragmentation index**	Dietary restraint	0.074 ± 0.036	0.039	0.074 ± 0.036	0.041
	Disinhibition	−0.027 ± 0.024	0.262	−0.026 ± 0.024	0.280
	Tendency towards hunger	0.027 ± 0.021	0.199	0.030 ± 0.022	0.172
**Sleep timing SD**	Dietary restraint	0.001 ± 0.010	0.952	0.005 ± 0.010	0.627
	Disinhibition	−0.000 ± 0.007	0.952	0.002 ± 0.007	0.744
	Tendency towards hunger	0.007 ± 0.006	0.259	0.007 ± 0.006	0.232
**Bedtime SD**	Dietary restraint	0.005 ± 0.009	0.590	0.008 ± 0.009	0.338
	Disinhibition	−0.001 ± 0.006	0.866	0.002 ± 0.007	0.777
	Tendency towards hunger	0.002 ± 0.005	0.745	0.002 ± 0.005	0.742

Data are reported as β coefficient plus standard error (SE). ^a^ Univariate linear regressions, unadjusted. ^b^ Multivariable linear regressions adjusted for age, race/ethnicity, sex, and education.

**Table 3:** Results of stratified analyses of sleep with eating behavior traits following significant interactions with sex.

Predictor	Outcome	*p*-Value (Interaction)	Women		Men	
			β ± SE	*p*-Value	Β ± SE	*p*-Value
**Sleep efficiency**	Tendency towards hunger	0.098	−0.008 ± 0.036	0.830	−0.149 ± 0.055	0.009
**Sleep onset latency**	Tendency towards hunger	0.013	−0.015 ± 0.033	0.653	0.169 ± 0.072	0.020
**Sleep fragmentation index**	Tendency towards hunger	0.013	−0.015 ± 0.026	0.564	0.115 ± 0.037	0.003

Data are reported as β coefficient plus standard error (SE). Multivariable linear regressions adjusted for age, race/ethnicity, sex, and education.

## Data Availability

The data presented in this study are available on request from the corresponding author.

## Supplementary Materials

The following are available online at https://www.mdpi.com/2072-6643/13/3/852/s1, Supplemental Table S1: Baseline descriptive characteristics of the overall analytic sample and by BMI.
