# Supplementary material for: Actigraphy-Derived Sleep Is Associated with Eating Behavior Characteristics
Source: Nutrients. 2021 Mar 5;13(3):852. doi: 10.3390/nu13030852 (PMC8001707; doi:10.3390/nu13030852)
Supplement: Supplementary file 1 [file nutrients-13-00852-s001.pdf]

SUPPLEMENTARY MATERIAL

Actigraphy-derived sleep is associated with eating behavior characteristics

Rocío Barragán, Faris M. Zuraikat, Victoria Tam, Samantha Scaccia, Justin Cochran, Si Li, Bin Cheng and Marie-Pierre St-Onge

Table S1. Baseline descriptive characteristics of the overall analytic sample and by BMI.

Characteristic	BMI<25 (N = 63)	BMI≥25 (N = 116)	Total (N = 179)	P-value
**Demographic**				
Age (years)				
Mean ± SD	32.3 ± 11.9	37.6 ± 13.4	35.7 ± 13.1	**0.009**
Range	19.0 - 65.0	19.0 - 73.0	19.0 - 73.0	
Sex				
Female	48 (76.2%)	75 (64.7%)	123 (68.7%)	0.112
Male	15 (23.8%)	41 (35.3%)	56 (31.3%)	
Race/Ethnicity				
White/Non-Hispanic	49 (77.8%)	81 (69.8%)	130 (72.6%)	0.255
Non-white/Hispanic	14 (22.2%)	35 (30.2%)	49 (27.4%)	
Education				
< College degree	24 (38.1%)	34 (29.3%)	58 (32.4%)	0.230
≥ College degree	39 (61.9%)	82 (70.7%)	121 (67.6%)	
**Health and Sleep Behaviors**				
Sleep duration				
Mean ± SD	435.8 ± 45.1	422.4 ± 48.2	427.1 ± 47.4	0.070
Range	316.9 - 553.1	204.9 - 527.4	204.9 - 553.1	
Bedtime				
Mean ± SD	23:41 ± 1:04	1:03 ± 5:13	1:09 ± 5:24	**0.007**
Range	20:10 – 2:06	21:56 – 2:49	20:10 - 2:40	
Waketime				
Mean ± SD	7:55 ± 1:10	7:55 ± 1:14	7:55 ± 1:13	0.625
Range	4:57 – 10:40	3:57 – 11:03	3:57 – 11:03	
Midpoint of sleep				
Mean ± SD	3:59 ± 00:58	4:01 ± 0:59	4:01 ± 0:59	0.311
Range	1:23 – 6:25	1:51- 6:59	1:23: – 6:59	
Sleep efficiency (%)				

Mean ± SD	89.0 ± 6.0	87.4 ± 6.0	87.9 ± 6.0	0.090
Range	67.5 - 97.1	67.0 - 97.0	67.0 - 97.1	
Wake after sleep onset (min)				
Mean ± SD	49.5 ± 29.1	54.8 ± 27.8	53.0 ± 28.6	0.234
Range	13.3 – 175.8	10.3 – 169.6	10.3– 175.8	
Sleep fragmentation index				
Mean ± SD	26.3 ± 8.8	27.4 ± 8.2	27.0 ± 8.4	0.369
Range	10.8 - 54.7	8.1 - 52.5	8.1 - 54.7	
Sleep duration SD				
Mean ± SD	53.3 ± 27.1	51.2 ± 31.4	51.9 ± 29.9	0.650
Range	12.6 - 140.6	4.0 - 201.2	4.0 - 201.2	
Bedtime SD				
Mean ± SD	60.8 ± 31.1	60.8 ± 37.3	60.8 ± 35.2	0.994
Range	20.3 - 174.1	0.0 - 240.0	0.0 – 240.0	
**Eating Behavior Traits**				
Dietary restraint				
Mean ± SD	6.5 ± 3.7	8.1 ± 4.1	7.5 ± 4.0	**0.015**
Range	0.0 - 16.0	0.0 - 19.0	0.0 - 19.0	
Disinhibition				
Mean ± SD	3.1 ± 2.3	4.0 ± 2.8	3.7 ± 2.7	**0.042**
Range	0.0 - 10.0	0.0 - 13.0	0.0 - 13.0	
Tendency towards hunger				
Mean ± SD	2.9 ± 1.8	3.1 ± 2.6	3.0 ± 2.4	0.596
Range	0.0 - 10.0	0.0 - 13.0	0.0 - 13.0	

Values are mean±SD for continuous variables and count (%) for categorical variables. BMI: body mass index; P-value for the comparisons (means or %) between BMI≥25 kg/m^2 and BMI<25 kg/m^2. Student's t test was used to compare continuous variables and Chi squared tests were used to compare categorical variables.
